# Brazos Valley Medical Association

**Published:** 1904-10

**Authors:** W. B. Briggs, E. N. Shaw

**Affiliations:** Secretary and Treasurer; Easterly, Texas; President; Easterly, Texas


President.

---

### Brazos Valley Medical Association.

Easterly, Texas, September 2, 1904.

The eighteenth semi-annual meeting of the Brazos Valley Medical Association will be held at Franklin, Texas, on the second Tuesday and Wednesday (8th and 9th) of November next. You are earnestly requested to prepare a paper on a theme of your own selection. You will have ample time. Please do not delay in this matter which is of such great importance to our grand order. We need your aid, or we would not ask it.

Send your subject to Dr. W. B. Briggs at once, as we desire to have programs out by October 1st. Don't fail to attend, as very important business is booked for this meeting.

W. B. Briggs, Secretary and Treasurer

